# Dynamic ultrasound molecular‐targeted imaging of senescence in evaluation of lapatinib resistance in HER2‐positive breast cancer

**DOI:** 10.1002/cam4.6607

**Published:** 2023-10-04

**Authors:** Xiaoyu Chen, Ying Li, Zhiwei Zhou, Yanqiu Zhang, Luchen Chang, Xiujun Gao, Qing Li, Hao Luo, Kenneth D. Westover, Jialin Zhu, Xi Wei

**Affiliations:** ^1^ Department of Diagnostic and Therapeutic Ultrasonography Tianjin Medical University Cancer Institute and Hospital, National Clinical Research Center for Cancer, Key Laboratory of Cancer Prevention and Therapy, Tianjin's Clinical Research Center for Cancer Tianjin China; ^2^ Department of Ultrasound Tianjin Hospital Tianjin China; ^3^ Breast Cancer Center Tianjin Medical University Cancer Institute and Hospital, National Clinical Research Center for Cancer, Key Laboratory of Cancer Prevention and Therapy, Tianjin's Clinical Research Center for Cancer Tianjin China; ^4^ Department of Radiation Oncology and Biochemistry University of Texas Southwestern Medical Center Texas Dallas USA; ^5^ School of Biomedical Engineering and Technology, Tianjin Medical University Tianjin China; ^6^ Cancer Center Daping Hospital, Third Military Medical University Chongqing China

**Keywords:** cellular senescence, HER2+ breast cancer, lapatinib resistance, senescence evasion, ultrasound‐targeted imaging

## Abstract

**Background:**

Prolonged treatment of HER2+ breast cancer with lapatinib (LAP) causes cellular senescence and acquired drug resistance, which often associating with poor prognosis for patients. We aim to explore the correlation between cellular senescence and LAP resistance in HER2+ breast cancer, screen for molecular marker of reversible senescence, and construct targeted nanobubbles for ultrasound molecular imaging to dynamically evaluate LAP resistance.

**Methods and Results:**

In this study, we established a new cellular model of reversible cellular senescence using LAP and HER2+ breast cancer cells and found that reversible senescence contributed to LAP resistance in HER2+ breast cancer. Then, we identified ecto‐5′‐nucleotidase (NT5E) as a marker of reversible senescence in HER2+ breast cancer. Based on this, we constructed NT5E‐targeted nanobubbles (NT5E‐FITC‐NBs) as a new molecular imaging modality which could both target reversible senescent cells and be used for ultrasound imaging. NT5E‐FITC‐NBs showed excellent physical and imaging characteristics. As an ultrasound contrast agent, NT5E‐FITC‐NBs could accurately identify reversible senescent cells both in vitro and in vivo.

**Conclusions:**

Our data demonstrate that cellular senescence‐based ultrasound‐targeted imaging can identify reversible senescence and evaluate LAP resistance effectively in HER2+ breast cancer cells, which has the potential to improve cancer treatment outcomes by altering therapeutic strategies ahead of aggressive recurrences.

## Graphical abstract

This st**udy f**irstly found that reversible senescence contributed to LAP(lapatinib) resistance in HER2+ breast cancer and identified ecto‐5′‐nucleotidase (NT5E) as a marker of reversible senescence in HER2+ breast cancer. We construct NT5E targeted nanobubbles (NT5E‐FITC‐NBs) as a new molecular imaging modality which could both target reversible senescent cells and be used for ultrasound imaging. Cellular senescence‐based ultrasound targeted imaging could identify reversible senescence early and evaluate LAP resistance effectively in HER2+ breast cancer.

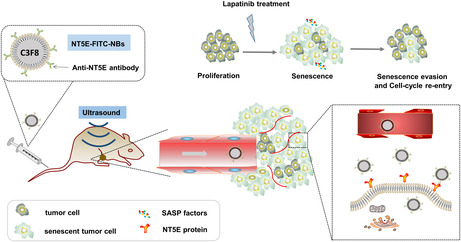

## 1 INTRODUCTION

Breast cancer has surpassed lung cancer as the most common cancer in female and is the most common type of cancer with the highest incidence and death rate of all cancers in 2020.^1^ About 20% of breast cancer cases are human epidermal growth factor receptor 2 positive (HER2+).^2^ HER2 (ErbB‐2) is a member of the epidermal growth factor receptor family, playing a key role in cell proliferation, survival, migration, and angiogenesis.^3^ HER2 amplification is often associated with a poor prognosis for breast cancer patients.^4^ The development of HER2‐targeted therapy has been one of the greatest advances in breast cancer treatment, including monoclonal antibodies and tyrosine kinase inhibitors (TKIs), which significantly improve the therapeutic outcome in clinic.^5^ Lapatinib (LAP) is HER2‐directed small molecule inhibitor used in the treatment of metastatic and recurrent HER2+ breast cancer.^6^ Notably, HER2+ breast cancer patients usually develop disease progression after certain time upon treatment with LAP due to the primary or acquired resistance.^7^ Therefore, to improve LAP therapeutic outcome, it is important to explore the mechanism of drug resistance to HER2‐targeted therapy.

Cellular senescence is an adaptive response to environmental stresses or injury characterized by stable exit from the cell cycle.^8^ Senescent cells lose the ability to proliferate, migrate, and invade. It has long been accepted that senescence is a tumor suppressive mechanism that permanently prevents cells at risk for malignant transformation. Therefore, the anti‐tumor effect induced by cellular senescence has been listed as a cancer treatment strategy.^9^ It has been shown that LAP induces reversible senescence in sensitive HER2+ cell lines.^7^, ^10^ However, senescent tumor cells are not permanently in a growth‐arrested state. Some solid tumor cells entered the phase of cellular senescence rather than apoptosis after treatment, and the senescent tumor cells stop dividing to avoid abnormal mitosis and escape from the cell killing effect of drugs; then, the secretion of cytokines in senescent cells can stimulate cell proliferation and tumor recurrence.^8^, ^11^ In some cases, senescent tumor cells can evade growth arrest and re‐enter the cell cycle when treatment withdrawn and these cells exhibit more aggressive growth phenotypes and higher tumor‐initiating capacity.^12^ In the present study, we hypothesize that cellular senescence promotes drug resistance and cancer relapse which plays an important role in LAP resistance in HER2+ breast cancer. Early detection of reversible senescence has the potential to improve cancer treatment outcomes by allowing clinicians to respond before tumors recur aggressively.

Therefore, it is critical to identify the molecular markers of reversible senescence which indicating LAP resistance. Ecto‐5′‐nucleotidase (NT5E/CD73), encoded by the *NT5E* gene, is a 70kD glycosylphosphatidylinositol (GPI) protein anchored on cell surface. Ample evidence has shown that NT5E is overexpressed in many cancers, such as breast cancer,^13^ pancreatic ductal adenocarcinoma,^14^ and non‐small cell lung cancer.^15^ Furthermore, NT5E has been found to be linked to the clinical characteristics and prognosis of cancer patients and plays a critical role during tumor progression.^16^ In detail, NT5E extensively involves multiple biological functions represented by growth, metastasis, angiogenesis, and drug resistance.^17^ In addition, NT5E (CD73) widely exists in various types of tumor microenvironment (TME) cells, and it can promote the tumor via crosstalk between the tumor cell and TME.^18^ At present, only a few studies investigated NT5E in breast cancer. In present study, we showed that NT5E was a LAP‐induced senescence marker that was highly expressed on the surface of senescent cells.

It is urgent to find an effective method to dynamically monitor NT5E, which reflects the effect of LAP therapy for breast cancer in an early and timely manner. The development of molecular imaging provides a new way to address this clinical problem, which can noninvasively identify more subtle changes in the tumor at the molecular level, before detectable anatomic or phenotypic changes have developed by analyzing the membrane proteins, secreted proteases, receptors, and other molecular biomarkers of tumor cells.^19^, ^20^ Ultrasound molecular‐targeted imaging is a kind of molecular imaging. Decoration of shell of ultrasound contrast agents with special molecules (ligands) can assist their binding to specific biomarkers that are overexpressed on targeted cells and assist their accumulating in target tissues specifically, so as to display the imaging changes at the molecular level in the target tissue and be convenient for us to better grasp the information of disease progression and treatment effect.^21^, ^22^ The use of ligands on the shell of ultrasound contrast agents to direct binding to target tissues for various purposes is now well established. In previous research, we have constructed poly(lactic‐co‐glycolic acid)‐poly(ethylene glycol)‐carbonic anhydrase IX mono antibody nanobubbles(PLGA‐PEG‐mAb_C__A__I__X_ NBs), which could detect early events in breast cancer progression,^23^ indicating that the ultrasound molecular‐targeted imaging is practical molecular diagnostic tool.

**FIGURE 1:**
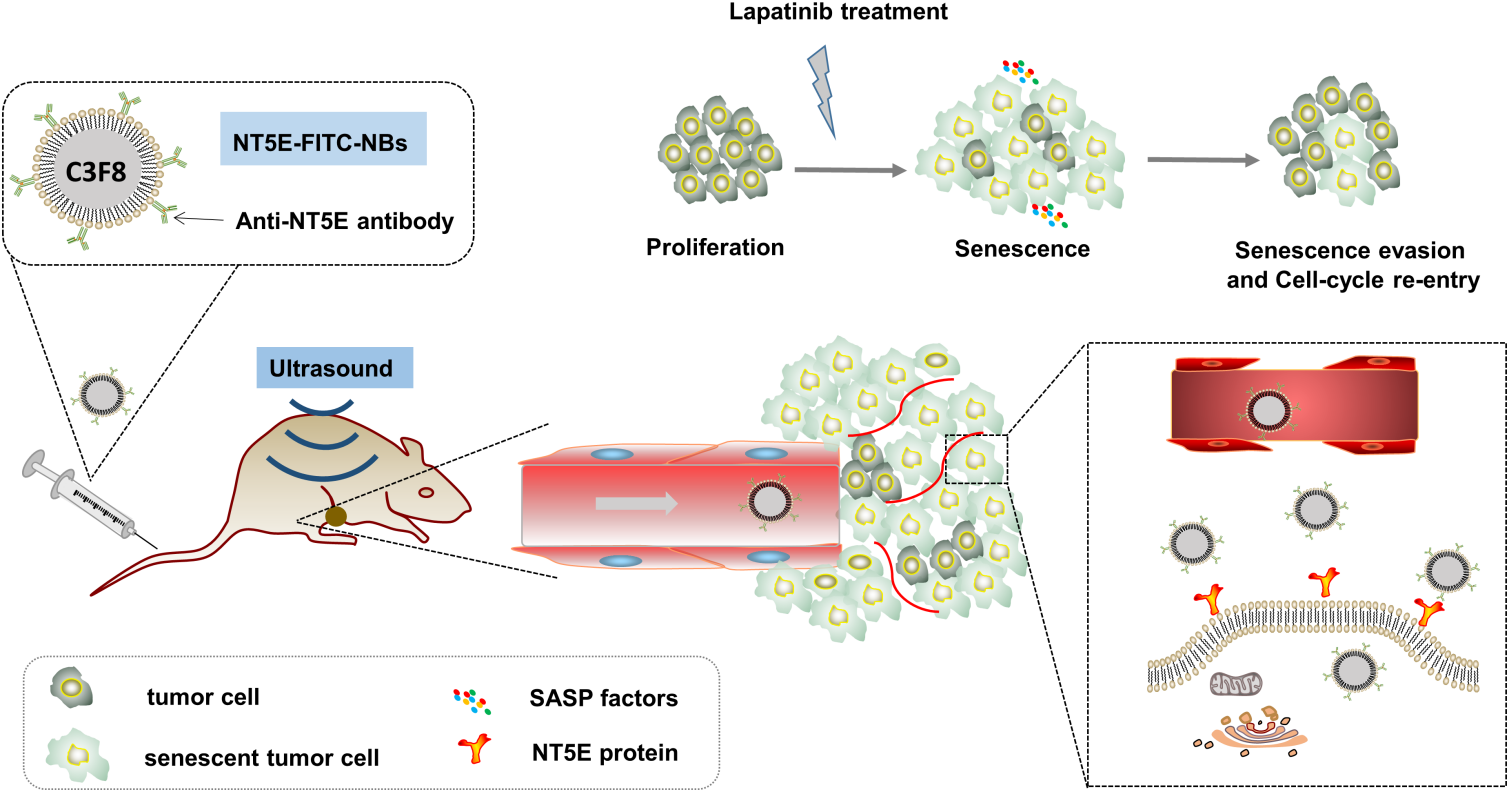
Schematic diagram of senescence detection using nanobubbles in LAP treated breast cancer. A brief elucidation for constructing targeted nanobubbles for ultrasound molecular imaging and dynamic evaluation of LAP resistance by targeting NT5E. LAP, lapatinib; SASP, senescence‐associated secretory phenotype.

In the present study, we used a senescence breast cancer cell model to identify potential molecular imaging targets, based on gene expression. Of the targets identified, we selected NT5E to develop a new ultrasound molecular imaging modality designed to detect reversible senescence in cancer. We use this new tool to identify cellular senescence in vivo. As shown in a schematic diagram (Figure 1).

## 2 MATERIALS AND METHODS

### 2.1 Cells and reagents

Human breast cancer cell line SKBR3 and HEK293T cells were purchased from ATCC. Both cell lines were cultured in DMEM medium (Gibco) with 10% fetal bovine serum (Gibco) at 37°C in a humidified atmosphere with 5% CO_2_. Routine authentication procedures were performed via implementing Short Tandem Repeat (STR) profiling, and all cell lines used in the present study were tested negative for Mycoplasma using Mycoplasma Plus PCR Primer Set (Agilent). Lapatinib (LAP) (Cat#: S2111, Selleckchem) and puromycin (Cat#: S7417, Selleckchem) were used in this study.

### 2.2 Plasmid and transfection

Plvx‐CMV promoter‐mCherry‐puro and plvx‐CMV promoter‐fluc‐puro plasmids (Han Hen) were obtained and modified by molecular cloning approaches to construct required plasmids: Plvx‐NT5E promoter‐mCherry‐puro and plvx‐NT5E promoter‐fluc‐puro. The resultant constructs were sequenced and confirmed then used for virus parking in HEK293T cells. After transfection for 48 h in HEK293T cells, viral supernatants were collected and used to infect target cells (SKBR3). After 48 h infection, SKBR3 cells underwent puromycin (2 μg/mL) selection for 2 weeks to obtain stable cell lines (SK‐NT5E promoter‐mCherry and SK‐NT5E promoter‐fluc).

### 2.3 CCK‐8 assay

Cell proliferation was assessed using Cell Counting Kit‐8 (US EVERBRIGHT) following the manufacturer's instructions. In brief, 5000 SKBR3 cells (100 μL) were seeded into 96‐well plates and treated with LAP over 72 h. After drug treatment, the medium was aspirated and cell proliferation was assessed with the addition of CCK‐8 working solution. The absorbance was measured at 450 nm with a microplate reader (BioTek) after 2‐h incubation in the dark.

### 2.4 SA‐β‐gal staining

Cell senescence was examined using Senescence β‐Galactosidase Staining Kit (CST, 9860S) following the manufacturer's instructions. Briefly, the medium was aspirated, and 1 mL fixation solution was added to fix the cells after drug treatment. SA‐β‐Gal staining solution was prepared and added, followed by an overnight incubation in a dry incubator at 37°C. Cell senescence was examined under a microscope by visualizing blue staining. All experiments were performed in triplicate.

### 2.5 Propidium iodide (PI) staining

Cell cycle was examined via PI staining. In brief, cells were collected and washed, and then fixed in 70% ethanol for 24 h. Subsequently, cell samples were stained with PI (50 μg/mL) (Tiangen) in the dark for 30 min at 4°C, and the cell cycle distribution was assayed by flow cytometry (BD Biosciences) and analyzed by ModFit software (Verity Software House).

### 2.6 Immunofluorescence (IF)

IF was performed to examine the protein expression and localization in SKBR3 cells. Briefly, cells were seeded onto cover slides in 24‐well plates. After treated with LAP, cells were washed with PBS and fixed with 4% paraformaldehyde for 15 min at room temperature, followed by 1‐h blocking with 5% BSA at room temperature. Subsequently, the corresponding primary antibodies were utilized to probe protein expression and localization overnight at 4°C. The next day, the cells were washed with PBS and incubated with fluorescent secondary antibody (FITC/Cy3, 1:500) for 1 h at room temperature in the dark. Then, the cells on cover slides were stained with DAPI and sealed onto the cell slides. The primary antibodies used were as follows: Anti‐γH2AX antibody (Cat#: 9718, 1:200, CST), Anti‐53BP1 antibody (Cat#: 88439, 1:400, CST), and Anti‐NT5E antibody (Cat#: ab133582, 1:100, Abcam).

### 2.7 RNA extraction and real‐time PCR

Total RNA from cells was extracted using the TRIzol Reagent (Invitrogen) according to the manufacturer's instructions. Total RNA was reverse transcribed into cDNA using the Prime Script™RT Master Mix (TakaRa). The resultant cDNA was used for subsequent real‐time PCR with the TB Green Premix Ex Taq II (TakaRa). Reactions, amplifications, and readings were performed on the LightCycler480 System (Roche). Primers for real‐time PCR were summarized in Table S1.

### 2.8 Colony formation assay

Colony formation assay was performed to test the ability of a single SK, SK‐T, and SK‐R cell to propagate to a colony. The cells were pretreated at the requirement of experimental design. A certain number of cells were inoculated into each well of 6‐ or 24‐well plate. After the single cell formed cell masses (10–30 days), the medium was aspirated and cells were fixed at room temperature with 4% paraformaldehyde for 20 min, followed by crystal violet staining for 30 min at room temperature. The cells were washed with water and dried for photography and counting.

### 2.9 RNA‐Seq

SKBR3 cells, senescent SKBR3 cells, and senescence escaped SKBR3 cells (three independent replicates for each cell) were lysed using TRIzol reagent (Invitrogen). Total RNA was isolated following the TRIzol‐based RNA extraction method and quantified using NanoDrop ND‐1000 (NanoDrop). cDNA libraries were generated and sequenced on an Illumina NovaSeq 6000 platform. The detailed information for mRNA library construction, sequencing, and preliminary analysis was provided in Appendix S1. Sequencing data were deposited in the Gene Expression Omnibus (GSE237413).

### 2.10 Kaplan–Meier analyses

The KM plotter online tool was used to investigate the mRNA expression of NT5E and its correlations with patient survival outcomes in different breast cancer subtypes. Parameters for cutoffs were *p* < 0.05 and hazard ratios excluding a value equal to 1.0.

### 2.11 Western blotting

Western blotting assay was performed as described previously.^24^ Cells were lysed for protein extraction, and protein concentration was measured using BCA Protein Assay Kit (Solarbio). Thereafter, protein samples were separated by SDS‐PAGE gels and transferred to PVDF membranes (Millipore). After blocking with 5% non‐fat milk for 1 h at room temperature, the membranes were incubated with primary antibodies at 4°C overnight and HRP‐conjugated secondary antibodies for 1 h at room temperature in the following day. Chemiluminescence was detected using a Bio‐Rad ChemiDoc XRS+ System. Bio‐Rad Image Lab software was used for densitometric analysis. The primary antibodies were as follows: anti‐THBS1 antibody (Cat#: ab267388 1:1000, Abcam), anti‐KIAA1324 antibody (Cat#: GTX46044, 1:1000, Gene Tex), anti‐GAPDH antibody (Cat#: ab181602, 1:10000, Abcam), and anti‐NT5E antibody (Cat#: ab133582, 1:1000, Abcam).

### 2.12 Preparation and characterization of targeted NBs

Two antibody‐conjugated complete NBs were prepared: IgG‐FITC‐NBs and NT5E‐FITC‐NBs. Commercially available perfluorocarbon‐filled, lipid‐shelled NBs were provided by SunLipo NanoTech. Antibody information is as follows: anti‐NT5E antibody and anti‐IgG antibody (Cat#: ab125938, Abcam). To generate targeted complete NBs, 0.1 mM antibody (NT5E or IgG) was dissolved into 15 mL sodium carbonate buffer solution (pH = 9.43) at 4°C by gently stirring; meanwhile, 1 mM FITC was dissolved in 15 mL DMSO and the FITC solution was dropped into antibody solution at the antibody to FITC ratio of 1:10 at 4°C in the dark for 24 h. The resultant solution was dialyzed with distilled water for 4 days with water change twice a day in the dark at 4°C. FITC modified antibodies (FA for short) were obtained after freezing and drying for next process.

FA was dissolved in PBS (pH = 6.0) and stirred gently, and NHS‐PEG‐NHS (MW = 500 Da) was dissolved in deionized water. Then, FA solution was mixed into PEG solution at 35°C and reacted for 15 min. After reaction, the mixed solution was placed into an ultrafiltration tube (cutoff size at 3000 Da). The product was washed with deionized water twice, then resuspended, freezed, and dried to obtain the NHS‐modified FA (FA‐NHS for short). Finally, the NBs with primary amine groups on the surface were resuspended into deionized water. FA‐NHS was dissolved into deionized water and quickly added into the aqueous solution of NBs, stirred gently, and reacted in the dark for 4 h. After reaction, the solution was dialyzed with deionized water (cutoff size at 100 kDa). The NBs with surface modified FA were obtained, termed as FA‐NBs (IgG‐FITC‐NBs and NT5E‐FITC‐NBs) for short. The particle sizes and zeta potentials of NBs were characterized by a Zetasizer Nano ZS90 particle size analyzer (Malvern Instruments Inc.).

### 2.13 Detect targeting efficiency of NBs in vitro

After preparation of antibody‐conjugated complete NBs, the targeting efficiency was examined in cells. Briefly, cells were seeded onto cover slides in 24‐well plate and exposed to DMSO or LAP for 7 days to induce cell senescence. Then, cells were incubated with 350 μg/mL NT5E‐FITC‐NBs or IgG‐FITC‐NBs for 30 min at 37°C in 5% CO_2_ atmosphere. Following incubation, cells were washed with PBS and fixed with 4% paraformaldehyde for 15 min at room temperature. Mounting medium with DAPI was used to stain cell nuclei and seal the cover slides onto cell slides. Cells were analyzed by positive fluorescence microscope (Axio Imager A2; Zeiss). In addition, SKBR3 cells were seeded into 6‐well plates. Cells are treated with the same procedure as abovementioned, and the cells were incubated with 350 μg/mL NT5E‐FITC‐NBs or IgG‐FITC‐NBs for 30 min at 37°C in 5% CO_2_ atmosphere. Then, the cells were washed, trypsinized, and collected for flow cytometry (BD Biosciences). The results were analyzed by Flowjo V10 software (BD Biosciences).

### 2.14 In vitro imaging of NBs

Targeted and non‐targeted antibody‐conjugated complete NBs were placed in a 1% gel well model; the imaging effects of NBs in B‐mode and CEUS were observed by ultrasonic equipment (Toshiba Canon Aplio500) with a frequency of 5–12 MHz and mechanical index (MI) of 0.06. The echo intensity of the region of interest in ultrasound image was determined using a TCA software (A built‐in software of Aplio500).

### 2.15 HER2+ breast cancer cellular senescence animal model

All animal experiments were approved by the International Animal Care and Use Committee of Tianjin Medical University Cancer Institute and Hospital. SK‐NT5E promoter‐fluc (fluc, firefly luciferase) stable cell line was established and propagated. Cell suspension was prepared by adding pre‐cooled sterile Matrigel and PBS in equal proportion to ensure that the density of suspension was no <10^7^ cells/mL. Four‐ to six‐week‐old BALB/C‐nu mice (Si Bei Fu Biotechnology) were selected to construct senescence animal model of breast cancer. Cell suspension (100 μL) was injected into the unilateral mammary gland fat pad of nude mice. Tumor formation was examined twice a week. Treatment was initiated when the diameter of the tumor was about 5 mm. The animals were randomly divided into three groups with five mice in each group. The three groups were control group (Con), senescence group (Sen), and evasion group (Eva), respectively. LAP suspension was prepared in 0.5–1% sodium hydroxymethyl cellulose (CMC‐NA) solution. Mice in Sen and Eva group were given LAP orally gavage for 2 weeks at 100 mg/kg/day, and mice in Con group were given CMC‐Na solution orally for 2 weeks. The treatment was stopped after 2 weeks. Mice in Sen and Con group were selected for bioluminescence imaging and ultrasound imaging, and then were sacrificed. In Eva group, the animals were kept after drug withdrawal until the tumors grew. At this time, Bioluminescence imaging and ultrasound imaging were performed on mice in the Eva group, and then, they were sacrificed. The tumor volume was calculated according to the following formula: volume = length × (width)^2^/2. Tumor volume and body weight were recorded every 3 days. After the mice were sacrificed, the tumors, heart, liver, spleen, lung, and kidney were removed for hematoxylin and eosin (HE) and immunohistochemical (IHC) staining.

### 2.16 Bioluminescence imaging

The fluc substrate solution was prepared by dissolving D‐luciferin potassium salt (Beyotime Biotechnology) in sterile D‐PBS. Mice were intraperitoneally injected with 200 μL D‐luciferin potassium salt solution (15 mg/mL) followed by anesthesia in 2% isoflurane. After 10–20 min of injection, the luminescence reached the most stable plateau stage, and then, bioluminescence imaging was carried out by IVIS Spectrum Optical in vivo imaging System (Caliper Life Science).

### 2.17 Ultrasound molecular imaging

The nude mice were firstly anesthetized with intraperitoneal injection of 10% chloral hydrate. Targeted and non‐targeted antibody‐conjugated complete NBs were injected into mice via tail vein at a volume of 200 μL. Then, B‐mode and CEUS imaging of tumor were observed by ultrasonic equipment (Toshiba Canon Aplio500) with a frequency of 5–12 MHz and mechanical index (MI) of 0.06. Imaging was delayed for 6 min after injection of NBs through the tail vein to allow targeted NBs to bind onto the tumor surface. The echo intensity of tumor region in ultrasound image was determined using a TCA software (A built‐in software of Aplio500). The difference of echo intensity in each group was statistically compared.

### 2.18 Clinical samples

From January 2011 to December 2020, eight pairs of tumor tissue specimens from HER2+ breast cancer patient before and after LAP treatment were screened from the Department of Breast at Tianjin Medical University Cancer Institute and Hospital. This protocol was approved by the Ethics Committee of Tianjin Medical University Cancer Institute and Hospital, and patients' informed consent and signature were obtained.

### 2.19 HE and IHC staining

Both HE and IHC staining were performed as described previously.^24^ The primary antibodies used were as follows: anti‐Ki67 antibody (Cat#: 9027, 1:200, CST), anti‐Cleaved Caspase‐3 antibody (Cat#: 9664, 1:100, CST), anti‐γH2AX antibody (Cat#: 9718, 1:250, CST), and anti‐NT5E antibody (Cat#: ab133582, 1:100, Abcam). The expression level of Ki67, Cleaved Caspase‐3, γH2AX, and NT5E was scored by a semi‐quantitative scoring system based on staining intensity and distribution using the following equation^24^: histoscore = staining intensity × percentage of positive cells. Staining intensity was defined as follows: 0 = negative; 1 = weak; 2 = moderate; and 3 = strong. The positive percentage was defined as follows: 0 = 0%; 1 = 0–25%; 2 = 25–50%; 3 = 50–75%; and 4 = 75–100%.

### 2.20 Statistical analysis

Data in this study are expressed as mean ± SD, and statistical analysis was conducted by *t* test and non‐parametric test. The significance of *p* value was as follows: *p < 0.05, **p < 0.01, ***p < 0.001, and ****p < 0.0001. All data were analyzed using GraphPad Prism (Version 8.3.0).

## 3 RESULTS

### 3.1 Breast cancer cells escape from LAP‐induced senescence after drug withdrawn

**FIGURE 2:**
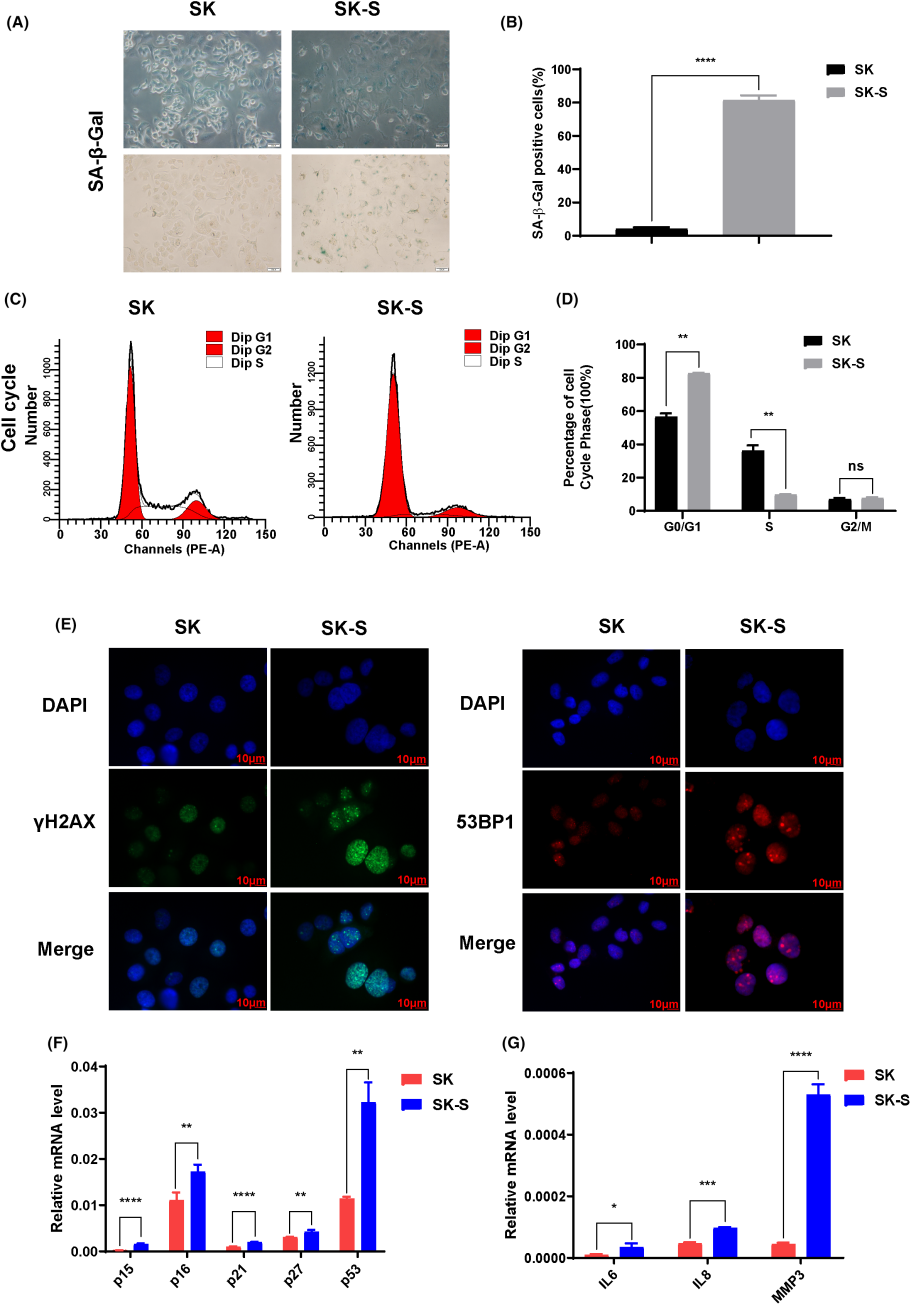
LAP induced cell senescence in HER2+ breast cancer cells. (A, B) Cell senescence development and morphology alteration in SK‐S cells, compared to parental SK cells. SK cells were treated with LAP at 250 nM for 7 days and subjected to SA‐β‐Gal staining. (C, D) Cell cycle distribution of SK and SK‐S cells determined by flow cytometry. (E) Expression of DNA damage foci in SK and SK‐S cells detected by immunofluorescence. γH2AX (green), 53BP1 (red), and nuclear DNA (blue) counterstained with DAPI (scale bar: 10 μm). Magnification: ×630. (F, G) Gene expression of senescence markers in SK and SK‐S cells measured by Real‐time PCR. SK, parental SKBR3 cells; SK‐S, senescent SKBR3 cells. *p < 0.05, **p < 0.01, ***p < 0.001,****p < 0.0001. Data are expressed as mean ± SD (*n* = 3).

Therapy‐induced cellular senescence has a multifaceted role in cancer therapy. It is a permanent blockade of cell proliferation on one hand; on the other hand, cellular senescence is associated with drug resistance and tumor recurrence. In order to evaluate the cell senescence in LAP treatment, we first assessed the effects of LAP on HER2+ breast cancer cell line, and SKBR3 cell line (herein referred to SK) was exposed to LAP at different concentrations for 24 h. The cell proliferation was assessed using CCK‐8 assay (Figure S1A). Growth of this cell line over a 2‐week period in 250 nM LAP caused an accumulation of senescent cells until 80% of cells were senescent by day 7 (Figure 2A,B and Figure S1B,C). Phenotypically, these cells were enlarged and flattened, and the cytoplasm was vacuolated, relative to parental SK cells, consistent with senescence (Figure 2A). Cells showing this effect are hereafter referred to as SK‐S (senescent SK cells).

To further confirm senescence in SK‐S cells, we performed cell cycle analysis and evaluated for senescence markers. SK‐S cells were mainly distributed in G0/G1 phase compared to SK cells (Figure 2C,D). IF staining showed a marked increase in the expression of γH2AX and 53BP1 in DNA damage foci of SK‐S cells relative to SK cells (Figure 2E). In addition, the expression senescence markers p15, p16, P21, p27, and p53 (Figure 2F) and senescence‐associated secretory phenotype (SASP) factors including IL6, IL8, and MMP3 were increased (Figure 2G).

**FIGURE 3:**
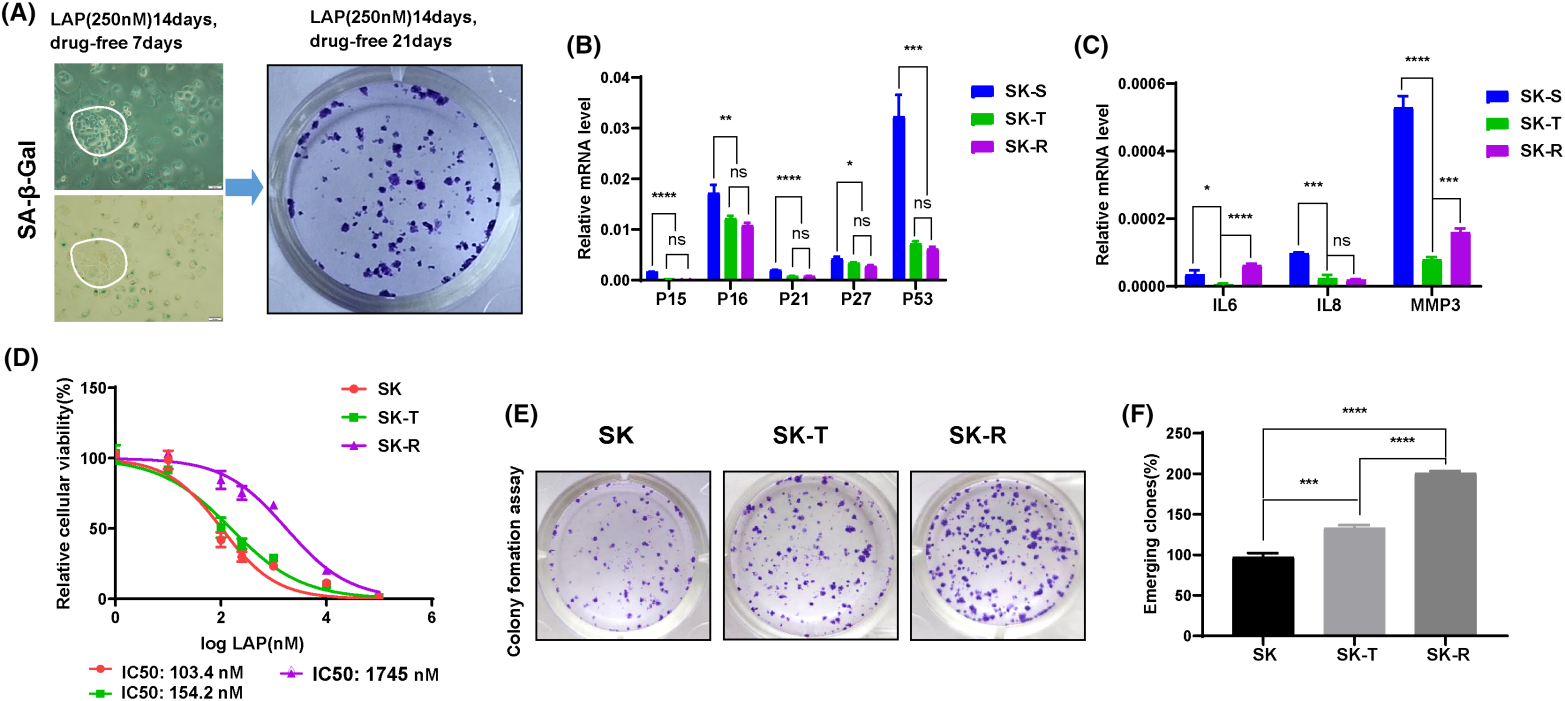
SK‐S cells evaded LAP‐induced senescence after drug withdrawal. (A) Senescent cell propagation after LAP withdrawal. SK cells were propagated in the presence of LAP at 250 nM for 14 days, followed by LAP withdrawn for 7 to 14 days, then subject to SA‐β‐Gal staining and clone formation assay. (B, C) Gene expression of senescence markers in SK‐S, SK‐T, and SK‐R cells detected by real‐time PCR. (D) Cell viability of SK, SK‐T, and SK‐R cells upon the exposure of LAP evaluated by CCK‐8 assay for 72 h. (E, F) Colony formation of SK, SK‐T, and SK‐R. LAP, lapatinib; SK, parental SKBR3 cells; SK‐S, senescent SKBR3 cells; SK‐T, senescence escaped SKBR3 cells; SK‐R, LAP‐resistant cells. *p < 0.05, **p < 0.01, ***p < 0.001, ****p < 0.0001. Data are expressed as mean ± SD (*n* = 3).

To test the hypothesis that LAP‐induced senescent cells can re‐enter cell cycle and proliferate, we evaluated the cell growth after LAP withdrawal. SK cells were induced senescence after 14‐day treatment of LAP at 250 nM, and the resultant SK‐S cells were maintain in LAP free culture condition for 7 days. The re‐proliferating cells among senescent cells were observed under light microscope, and the SA‐β‐Gal staining of these cells was negative (Figure 3A). Replating of these SK‐S cells over 21 days resulted in colony formation indicating that the senescent cells had re‐entered the cell cycle (Figure 3A). To further assess the changes in senescent biomarkers for these re‐proliferating cells, we selected the single, seclude colony and propagated. The resultant cells were identified as senescence escape cells and were referred as SK‐T.

Additionally, we generated LAP‐resistant cell line (herein referred as SK‐R) which was obtained when SK cells were exposed LAP at an escalating concentration from 250 nM up to 5 μM. In comparison with SK‐S cells, the SA‐β‐Gal staining of SK‐T and SK‐R was negative (Figure S2A,B) and the cell population in the G0/G1 phase was marked decreased in SK‐T and SK‐R cells (Figure S2C,D), indicating that SK‐T and SK‐R cells had re‐entered the division stage after senescence. In consistence with the SA‐β‐Gal staining and cell cycle distribution observations, the IF staining showed a remarkable decline in the protein expression level of γH2AX and 53BP1 in DNA damage foci of SK‐T and SK‐R cells (Figure S2E,F).

Real‐time PCR results displayed a dramatic decrease in the gene expression level of conventional senescence markers including *p15*, *p16*, *p21*, *p27*, and *p53* (Figure 3B) and SASP factors *IL6*, *IL8*, and *MMP3* in SK‐T and SK‐R cells, compared to SK‐S cells (Figure 3C). In order to determine whether the senescence escaped cells were resistant to LAP to a certain extent, we compared the IC_50_ of SK, SK‐T, and SK‐R cells using CCK‐8 assay (Figure 3D). It showed that SK‐R cells were the most unresponsive ones to LAP, to a lesser extent, SK‐T (Figure 3D), which indicates that senescence escaped cells enhance their resistance to LAP compared to parental cells. Subsequently, comparing the number of clones formed by SK, SK‐T, and SK‐R cells, it was found that the colony number of SK‐T was less than that of SK‐R, but more than that of SK, indicating that the cloning ability of senescence escaped cells was enhanced (Figure 3E,F). Together, it suggests that senescence escape could be one of the causes of drug resistance of cancer cells.

### 3.2 Screening and validation of cellular senescence markers

Following the identification of SK‐T and SK‐S cells, next attempt is to decipher the transcriptional difference in these cells. We performed RNA‐seq to evaluate the alterations in gene expression on three groups of cells, including SK, SK‐S, and SK‐T. It showed a marked alteration in the transcription profile of SK‐S and SK‐T cells, relative to SK cells. The Venn diagram showed 17,267 overlapping genes in SK, SK‐S, and SK‐T cells, including 814 genes in SK‐S and SK cells, 559 genes in SK‐T and SK cells, and 468 genes in SK‐S and SK‐T cells (Figure S3A). There were 722 unique genes for SK cells, 1182 unique genes for SK‐S cells, and 476 unique genes for SK‐T cells (Figure S3A). Further analysis for changes in the expression level revealed 1223 upregulated genes and 1081 downregulated genes in SK‐S compared to SK cells; 202 upregulated genes and 370 downregulated genes in SK‐T compared to SK cells; and 1171 upregulated genes and 1690 downregulated genes in SK‐T compared to SK‐S cells (Figure S3B). It suggests a differential alteration in the transcriptional profiles of SK‐S and SK‐T cells, relative to parental SK cells. Moreover, the exhibition on the heat map presented a total number of 7283 genes that differed significantly among the three groups of cells (Figure S3C). GO enrichment analysis and KEGG enrichment analysis were carried out for differentially expressed genes in the three groups of cells, respectively (Figure S3D). Then, Top20 GO term and Top20 Pathway with the smallest *p* value (the most significant enrichment) were selected from the enrichment analysis results to draw scatter diagram. GO analysis filtered terms related to cell response to DNA damage stimulation, cell cycle, DNA replication, and DNA repair, while KEGG enriched signal pathways concerning DNA replication, cell cycle, and cellular senescence. These results suggested that the different genes of the three groups of cells were involved in these biological processes and signaling pathways.

**FIGURE 4:**
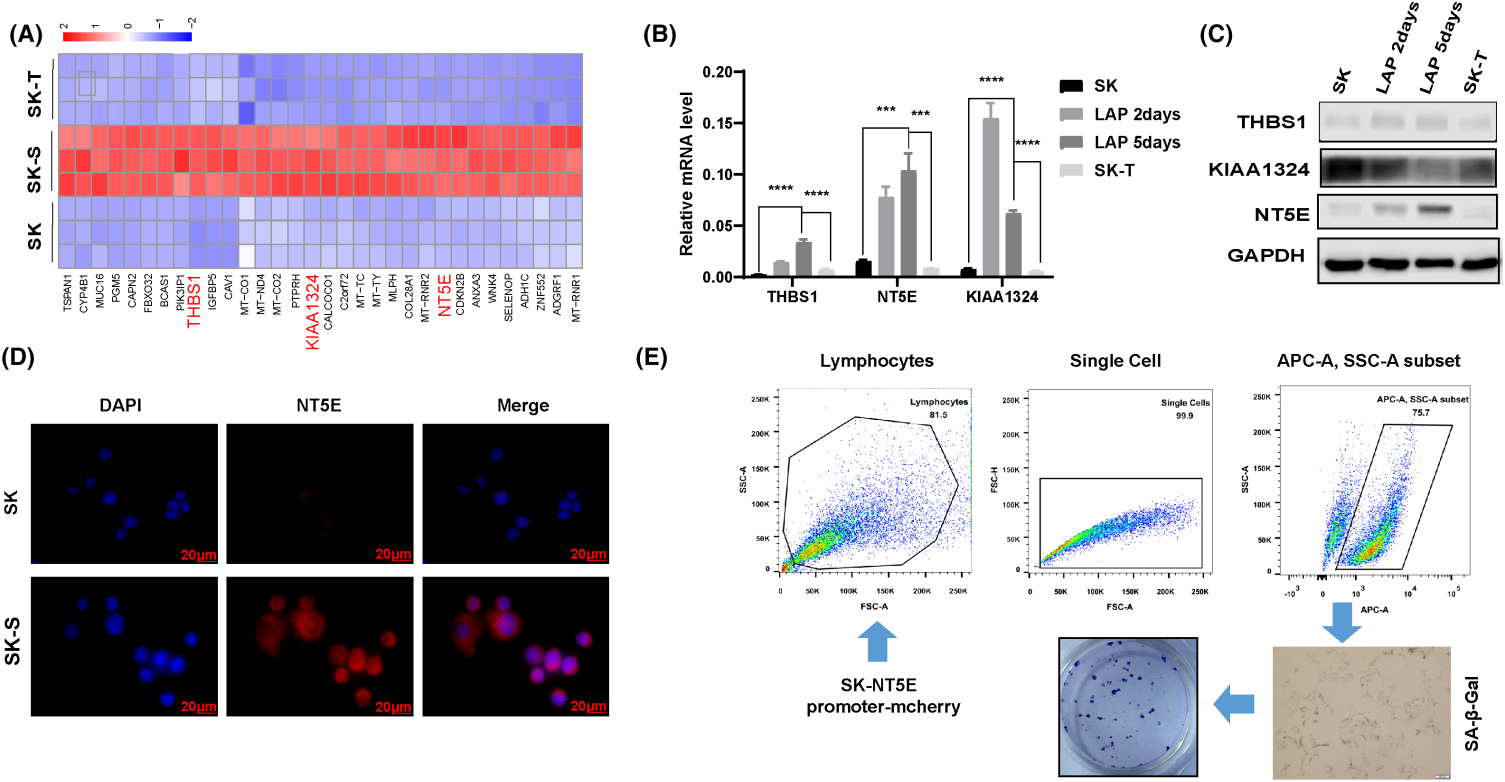
NT5E was identified as a cellular senescence marker. (A) Screened top 32 genes upregulated only in the senescent cells, which were from the top 100 genes with the lowest *p* value among the differentially expressed genes in the three groups were selected and displayed by heatmap. Senescence‐associated genes *THBS1*, *NT5E*, and *KIAA1324* were highlighted in red. (B) Gene expression profile of *THBS1*, *NT5E*, and *KIAA1324* in different context of SK cells. (C) Protein expression profile of *THBS1*, *NT5E*, and *KIAA1324* in different context of SK cells. (D) Cell membrane localization of NT5E protein examined by immunofluorescence. NT5E (red) and nuclear DNA (blue) counterstained with DAPI (scale bar: 20 μm). Magnification: ×400. (E) Development of verification of a stable cell line SK‐NT5E promoter‐mCherry. Senescent cells expressing red fluorescent protein mCherry were sorted out by flow cytometry, when senescence was induced by LAP in the stable cell line. The sorted cells were propagated and subject to SA‐β‐gal staining and colony formation assay. Fold change (FC) ≥ 2 or FC ≤ 0.5 and *p* < 0.05. SK, parental SKBR3 cells; SK‐S, senescent SKBR3 cells; SK‐T, senescence escaped SKBR3 cells; ***p < 0.001, ****p < 0.0001. Data are expressed as mean ± SD (*n* = 3).

To narrow in for screening senescence targets, we selected and displayed the top 32 genes upregulated only in the senescent group (SK‐S), which were from the top 100 genes with the lowest *p* value among the differentially expressed genes in three groups (Figure 4A). There were three genes (*THBS1*, *NT5E*, and *KIAA1324*) associated with cellular senescence and expressed on the cell membrane screened from the 32 genes, and their mRNA and protein expression were examined by real‐time PCR and Western blot, respectively (Figure 4B,C). It showed that with the extension of LAP treatment time, the proportion of senescent cells increased, leading to an increase in the mRNA level of the three genes, while in SK and SK‐T cells, the mRNA level decreased. However, only the protein expression of NT5E increased significantly, which was consistent with that of mRNA level. In addition, the IF staining also exhibited an increase in NT5E in SK‐S cells, relative to SK cells (Figure 4D). Therefore, NT5E was selected as the senescence target.

Previous experiments demonstrated that SK cells could escape from LAP‐induced senescence and reproliferate after drug withdrawal. In order to confirm whether the re‐proliferating cells were derived from senescent cells, we established a stable cell line with SK‐NT5E promoter‐mCherry which could report cellular senescence. When LAP induced senescence of stable cell line, NT5E promoter was activated and senescent cells expressing mCherry were sorted out by flow cytometry. The senescent cells were further cultured, and it was found that the sorted cells were positive for SA‐β‐Gal staining (Figure 4E). After 21–27 days, the sorted cells continued to propagate and form monoclonal clusters (Figure 4E). It was confirmed that escaped cells were indeed derived from senescent cells and NT5E could be an effective marker of cellular senescence.

### 3.3 Binding capacity of ultrasound‐targeted nanobubbles to senescent cells

**FIGURE 5:**
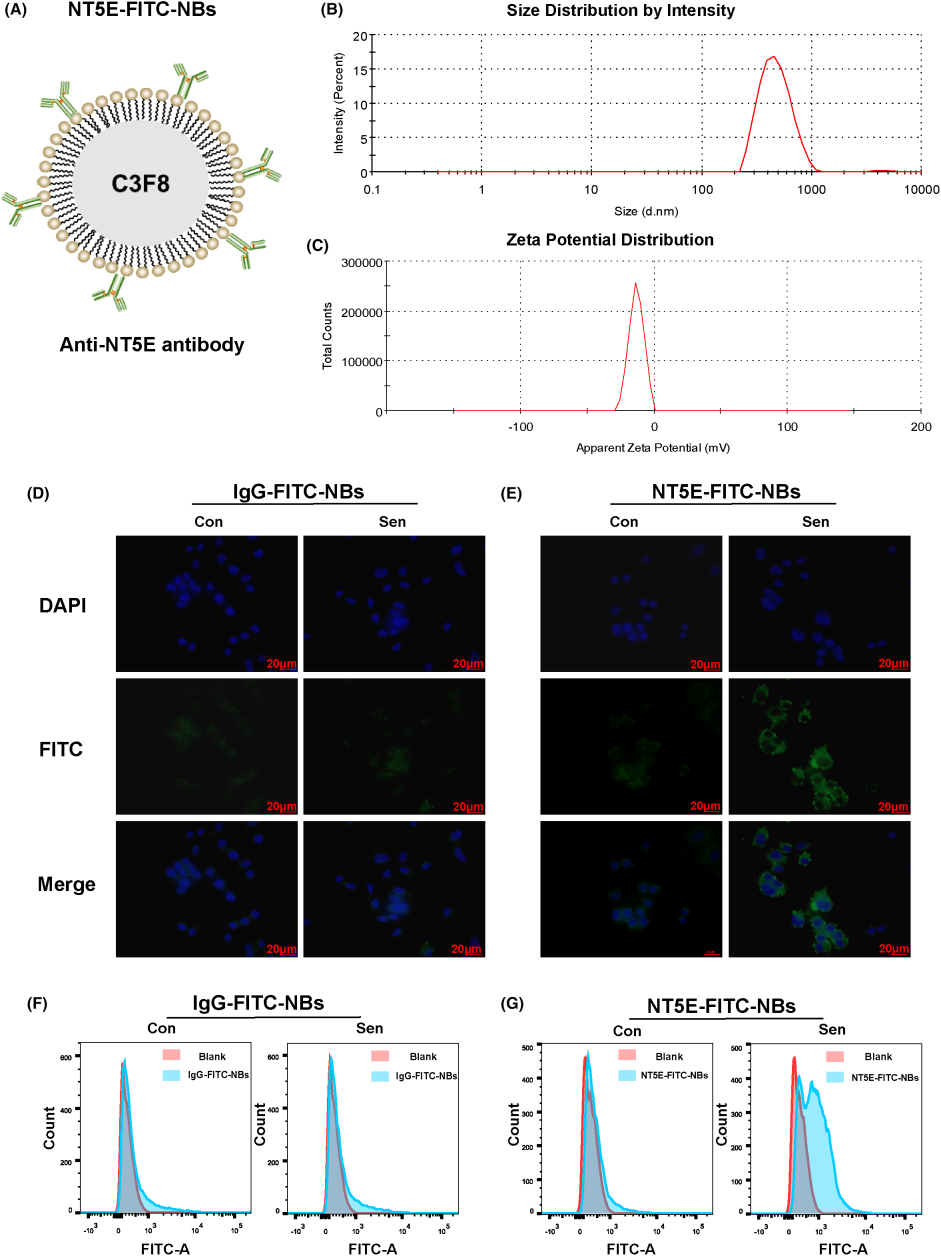
Ultrasound‐targeted nanobubbles bound to senescent cells. (A) Structure diagram of NT5E‐FITC‐NBs. (B, C) Particle size and Zeta potential of NT5E‐FITC‐NBs. (D) Fluorescence microscope image of tumor targeting and penetrating efficiency of IgG‐FITC‐NBs and NT5E‐FITC‐NBs in vitro after incubation for 30 min at 37°C in 5% CO_2_ atmosphere. The blue fluorescence was DAPI, and green fluorescence was NBs (scale bar: 20 μm). Magnification: ×400. (E, F) The binding ability of IgG‐FITC‐NBs and NT5E‐FITC‐NBs to senescent cells detected by flow cytometry. Con, control group; Sen, senescence group.

Next, we attempted to device a senescent detection strategy. Previously, we assembled nanobubbles to detect tamoxifen resistance in ER+ breast cancer.^23^ We would adapt this technique to device nanobubbles to detect LAP‐induced senescence in HER2+ breast cancer. Ultrasound nanobubbles had a core of perfluoropropane bubble (C3F8) and a shell of lipid (Figure 5A). NT5E antibody labeled with green fluorescence was coupled to the nanobubble shell to obtain NT5E‐FITC‐NBs with a particle size of 486.9 ± 8.2 nm and a zeta potential of −12.1 ± 10.3 mV (Figure 5B,C). Then, IgG antibody labeled with green fluorescence was coupled to the nanobubble shell to obtain IgG‐FITC‐NBs with a particle size of 438.0 ± 12.5 nm and a zeta potential of −10.2 ± 8.3 mV (Figure S4A,B). IgG‐FITC‐NBs was served as the control for NT5E‐FITC‐NBs. IgG‐FITC‐NBs and NT5E‐FITC‐NBs with different concentrations were incubated with SK cells for 12 and 24 h, respectively, and it was found that there was no statistical difference in cell activity at different concentrations of NBs (Figure S4C). In order to test the targeting ability of ultrasound nanobubbles to senescent cells (Figure 5D,E), it could be seen through IF that NT5E‐FITC‐NBs were obviously gathered around the senescent cell membrane and some entered the cytoplasm. Their binding rate to senescent cells was significantly higher than that of cells in the control group, while IgG‐FITC‐NBs had no significant fluorescence accumulation around the cells in the control group and senescence group. The results of flow cytometry were consistent with the above results (Figure 5E,F).

With the advantage of nanoscale particle size, NT5E‐FITC‐NBs could reach the tumor tissue through the leaky vasculature of the tumor. With the guidance of NT5E targeting, the accumulation of NT5E‐FITC‐NBs in tumors increased, and it was easier to penetrate the extracellular matrix and cell membrane into the cytoplasm. Subsequently, we tested the in vitro imaging effect of NT5E‐FITC‐NBs and IgG‐FITC‐NBs detected by ultrasonic equipment, and found that both nanobubbles had good imaging effects in B‐mode and CEUS (Figure S4C).

### 3.4 Evaluation of LAP‐induced senescence in HER2+ breast cancer by ultrasound‐targeted imaging

**FIGURE 6:**
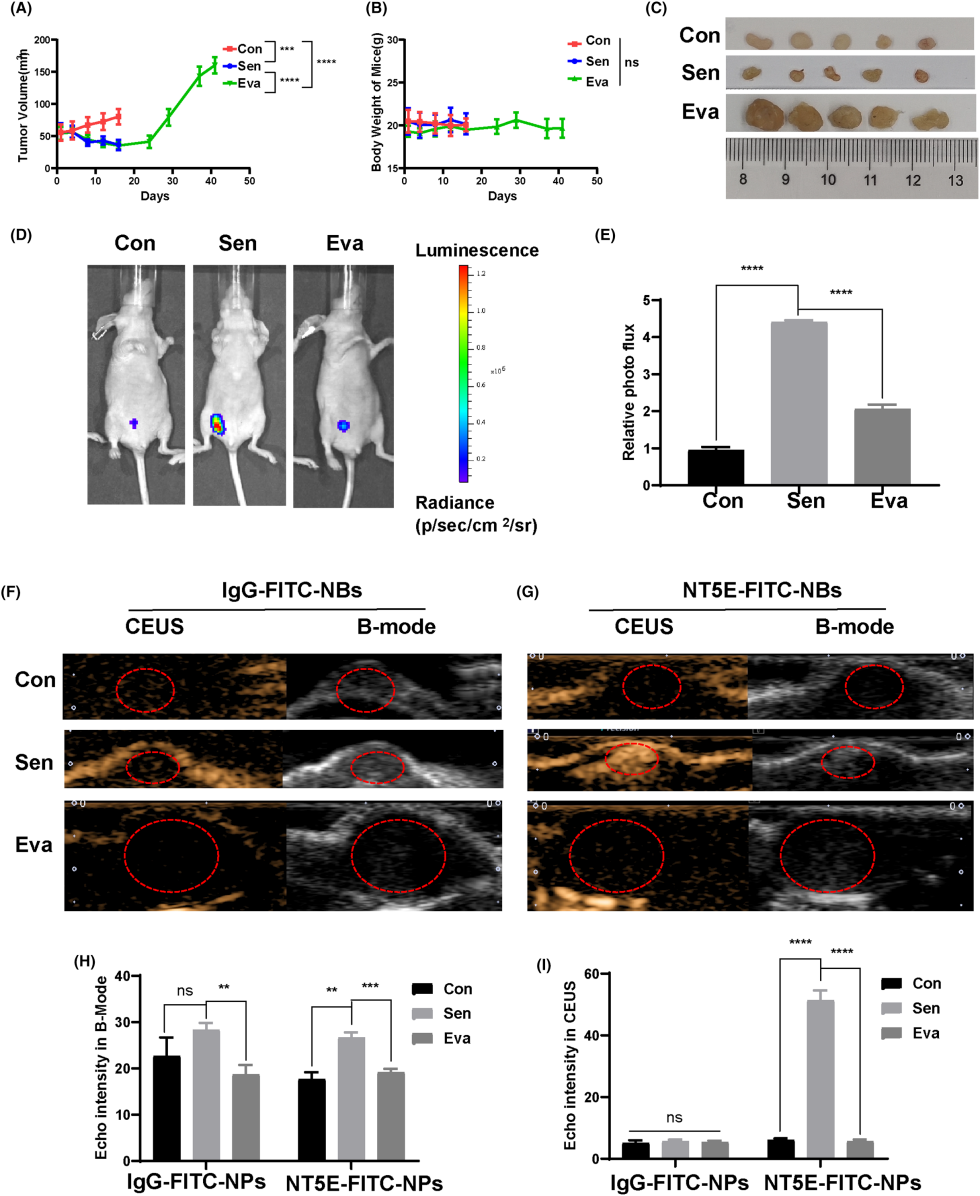
LAP‐induced senescence in HER2+ breast cancer was detected by ultrasound‐targeted imaging. (A, B) Alterations in tumor volume and body weight in Con, Sen, and Eva groups of mice. (C) representative image of tumor in Con, Sen, and Eva groups. (D, E) Validation of senescence animal model of breast cancer by bioluminescence imaging. (F–I) Ultrasound imaging of IgG‐FITC‐NBs and NT5E‐FITC‐NBs in three groups in vivo (B‐mode and CEUS). Con, control group; Eva, evasion group; Sen, senescence group. **p < 0.01, ***p < 0.001, ****p < 0.0001. Data are expressed as mean ± SD (*n* = 5).

To evaluate binding of targeted NBs to cellular senescence in vivo, a senescence animal model of breast cancer was employed. We constructed SK‐NT5E promoter‐fluc stable cell line, and the senescence animal model was established by injecting the stable cell line into mammary gland fat pad of female nude mice. LAP treatment can activate the NT5E promoter of tumor cells and make cells bioluminescent. As shown in Figure 6A–E, when mice were treated with LAP for some time, the tumor in Sen group shrank and the luminescence signal was enhanced, while the luminescence signal of Con group was relatively weak. After long drug withdrawal periods, the tumor in Eva group was enlarged, the luminescence signal was weakened.

After completing bioluminescence imaging, the three groups of animals were injected with IgG‐FITC‐NBs and NT5E‐FITC‐NBs via the tail vein for ultrasound imaging (Figure 6F, G). The changes in the signal intensity of the three groups of ultrasound imaging were clearly consistent with those of bioluminescence imaging. After animals were treated with LAP, the tumor shrank, the luminescence signal was enhanced, and the ultrasound echo intensity increased. After long drug withdrawal periods, the tumor was enlarged, the luminescence signal was weakened, and the ultrasound echo intensity decreased. Compared with the Con and Eva groups, the echo intensity of NT5E‐FITC‐NBs in the Sen group was the strongest in both B‐mode and CEUS, while the echo intensity of IgG‐FITC‐NBS was not statistically different among the three groups, which proved the effectiveness of NT5E‐FITC‐NBs for ultrasound imaging in the evaluation of cellular senescence induced by LAP in HER2+ breast cancer (Figure 6F–I).

**FIGURE 7:**
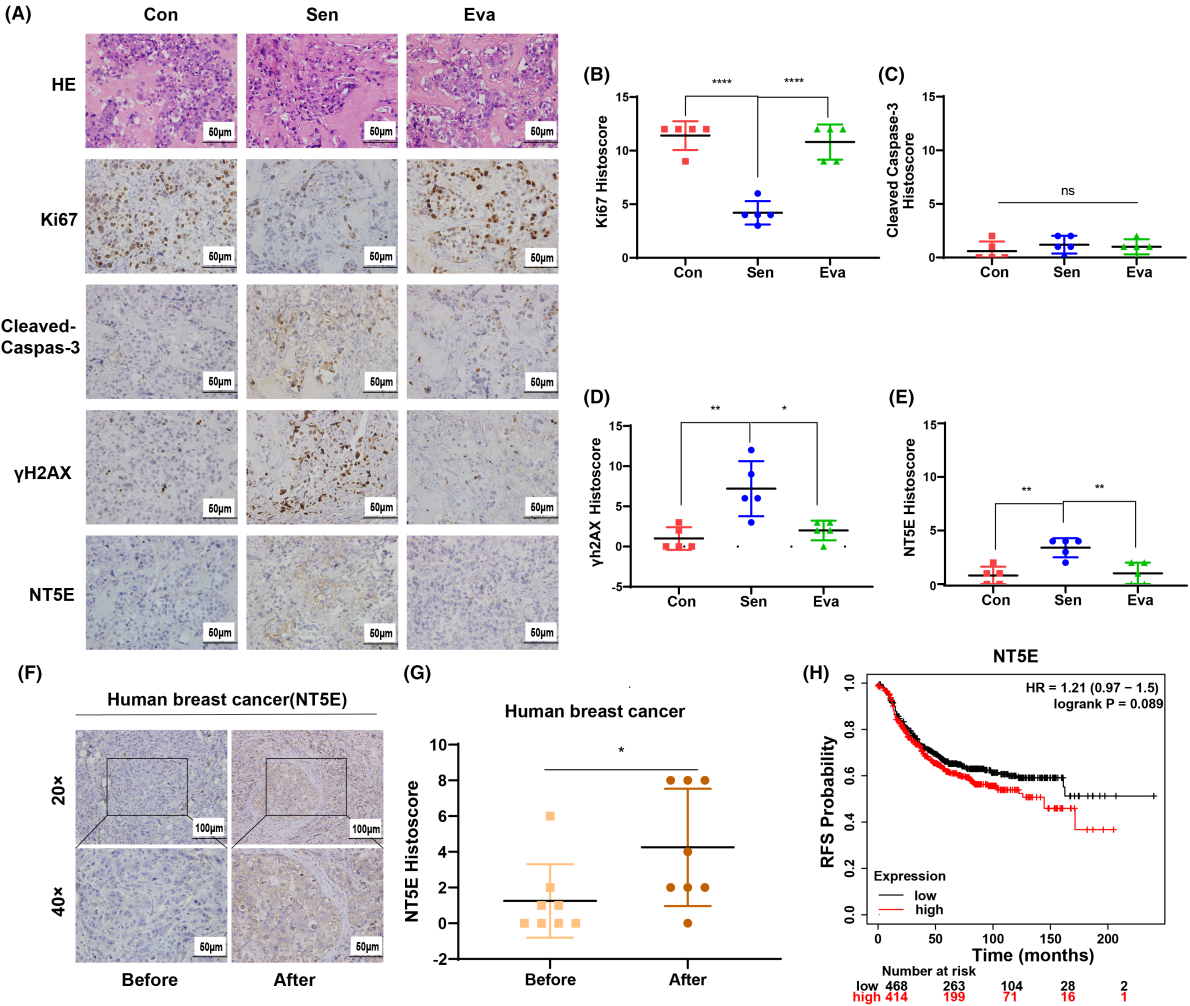
NT5E was a risk factor affecting the prognosis of breast cancer patients. (A–E) Representative images of HE and IHC staining and histoscore of Ki67, Cleaved Caspase‐3, γH2AX, and NT5E of tumors in Con, Sen, and Eva group. Magnification: ×400. Data are expressed as mean ± SD (*n* = 5). (F, G) Representative IHC images of NT5E and histoscore of eight pairs of tumor tissue specimens from HER2+ breast cancer before and after LAP treatment. Data are expressed as mean ± SD (*n* = 8). (H) Kaplan–Meier analysis for NT5E expression under the condition of RFS probability in patients with HER2+ breast cancer using K‐M Plotter database. Con, control group; Eva, evasion group; RFS, recurrence free survival; Sen, senescence group. Scale bar: 50 μm; *p < 0.05, **p < 0.01, ****p < 0.0001.

After ultrasound imaging, the nude mice were sacrificed, and the tumors were collected for HE and IHC staining. The results of IHC showed that (Figure 7A–E), compared with the Con group, the expression of Ki67 was significantly decreased in the Sen group, while the expression of Ki67 was increased in the senescence escaped Eva group. Furthermore, there was no statistical difference in Cleaved Caspase‐3 expression among the three groups, suggesting that the effect of LAP on HER2+ breast cancer was mainly to inhibit the proliferation of tumor cells, rather than to induce apoptosis. The expression of γH2AX and NT5E in Sen group was significantly higher than that in Con and Eva group, which was consistent with our cell experiment results, further proving that NT5E can be used as an effective marker of senescence and the effectiveness of NT5E‐FITC‐NBs for ultrasound‐targeted imaging. After ultrasound imaging, viscera (heart, liver, spleen, lung, and kidney) were taken from the mice for HE staining (Figure S5). It was found that there was no significant difference in morphology and structure of the heart, liver, spleen, lung, and kidney of the three groups of mice in HE staining, which suggested that the ultrasound‐targeted nanobubbles used in the experiment were tolerable and safe.

Subsequently, eight pairs of tumor tissue specimens from HER2+ breast cancer before and after LAP treatment were screened for NT5E IHC staining and scoring. It was found that after LAP treatment, the NT5E staining score of tumor tissue was higher than that prior to treatment, and the difference was statistically significant (Figure 7F,G). Notably, the data of 882 HER2^+^ breast cancer patients from K‐M Plotter database were retrieved and the survival curve RFS (recurrence free survival) analysis was conducted. The results showed that there was no statistical difference in RFS probability between the groups with high and low expression level of NT5E; however, the hazard ratio (HR) of the group with high expression of NT5E was >1, indicating that NT5E was a risk factor affecting the prognosis of HER2^+^ breast cancer patients (Figure 7H). Clinical data suggested that ultrasound‐targeted nanobubbles based on NT5E as a senescence target had the potential of clinical translation.

## 4 DISCUSSION

In this study, we have shown that prolonged treatment of HER2+ breast cancer with LAP causes cellular senescence in SKBR3 cells. SKBR3 cells have a mutation in p53. p53 has been reported to be essential for the establishment of oncogene‐induced senescence,^25^ while other studies have shown that therapy‐induced senescence (TIS) can occur independently of p53.^26^ SKBR3 cells carry functional mutations in p53, while HCC1419 cells are p53 deficient.^27^ It has been reported LAP‐induced senescence in both LAP sensitive HER2+ cell lines, suggesting that LAP‐induced senescence is not dependent on wild‐type p53 activity.^10^ Restoring wild‐type p53 activity either by transfection of wild‐type p53 into HCC1419 cells or by treatment SKBR3 cells with APR‐246,^28^ a molecule which reactivates wild‐type p53, blocked LAP‐induced senescence and caused increased cell death.^10^ These results suggest that low wild‐type or mutant p53 activity contributes to induction of LAP‐induced senescence, whereas wild‐type p53 may facilitate LAP‐induced apoptosis.

As stated above, breast cancer cells escaped from LAP‐induced senescence after drug withdrawn. Studies^29^ have confirmed that senescent cells can secrete SASP factors that mediate secondary effects on tumor progression. Different SASP factors contribute to cancer stemness, proliferation, migration, invasion, and metastasis, thus enhancing the malignant potential of the cancer cell population. Another important mechanism by which senescent cells and their SASP indirectly contribute to cancer progression and relapse is the negative modulation of the immune system. In the present study, IL6, IL8, and MMP3, which have been highlighted for their pro‐tumorigenic activities of individual SASP factors in many studies,^30^, ^31^ produced by LAP‐induced senescent breast cancer cells. In addition, we have demonstrated the transcriptional difference in different status of HER2+ breast cancer cell lines. There were significant alterations in the transcription profile for parental cells, senescent cells, and senescence escaped cells. The RNA‐seq results revealed the differential expression level of three genes participate in cellular senescent event, including *THBS1*, *NT5E*, and *KIAA1324*. Other genes with significant alterations in expression level in the three groups of cells were enriched in biological processes including cellular response to DNA damage stimulus, cell cycle, DNA replication, and DNA repair, etc. These biological processes may be involved in the senescence escape caused by the targeted therapy of LAP for HER2+ breast cancer. Therefore, we suggest that SASP mediate the evasion of senescent cells from LAP‐induced cellular senescence and the reasons for reactivation of proliferation by senescent cancer cells.

We identified NT5E as a marker of reversible senescence in HER2+ breast cancer. High expression of NT5E is generally associated with poorer clinical outcomes in various cancer.^32^ NT5E is involved in immune‐related drug resistance. NT5E enhances the effect of suppressive immune cells such as myeloid‐derived suppressor cells (MDSCs) and T regulatory cells (Tregs) and attenuates the protective immune cells including T cells and NK cells to weaken anti‐tumor immunity, through interaction with specific G‐protein‐coupled receptors A1, A2A, A2B, and A3.^33^, ^34^ Elham et al^35^ reported that NT5E, as a tumor‐intrinsic inhibitory immune checkpoint, can contribute chemotherapy resistance and facilitate non‐small cell lung cancer development. Turcotte et al^36^ showed that, in HER2+ breast cancer, NT5E expressed by tumor cells and host cells significantly suppresses the anti‐ErbB2‐mediated immune response, thereby promoting resistance to targeted therapy. In addition to its enzyme‐dependent function of producing immunosuppressive adenosine, NT5E also plays a critical role in engaging tumor plasticity involving stemness, epithelial to mesenchymal transition, metastasis, and treatment evasion for a wide range of solid tumor types.^32^ Adding to this background, several preclinical studies have promoted NT5E as a potential therapeutic target for cancer therapy. The synergistic combination of blockade of NT5E and conventional anticancer therapies (such as chemotherapy, radiotherapy, and immunotherapy) or targeted therapies can boost the efficacy of existing therapies, magnify their anti‐proliferation effect, and restore immune surveillance.^37^ This study further establishes NT5E as a clinically relevant marker in cancer, both for the development of diagnostic tools, but also as a prognostic marker. Therefore, NT5E‐based imaging may have potentials beyond monitoring reversible senescence.

A challenge for successful implementation and prediction of drug resistance resulted from senescence is that whether cells actually could escape from therapy‐induced senescence. It is possible that some tumor cells never undergo a stable senescence arrest before re‐entering the cell cycle. In order to address this question, we established a SK‐NT5E promoter‐mCherry stable cell line which enables us to explore whether escaped cells are derived from senescent cells. With the utilization of this stable cell line, we confirm that escaped cells are indeed derived from senescent cells and NT5E could be an effective marker of cellular senescence.

A key to molecular targeted ultrasound imaging is the selection of specific ligands which determine the location and application of targeted imaging. In present study, we showed that NT5E was a LAP‐induced senescence marker that was highly expressed in senescent cells. Moreover, NT5E was displayed on the cell surface, making it attractive as a molecular target for nanobubbles. It has been known that, in most scenario cases of cellular senescence, the conventional markers are intracellular protein which make them not suitable for this imaging approach. Since we have shown the important role of reversible senescence in LAP resistance in HER2+ breast cancer, in order to dynamically monitor and effectively evaluate LAP resistance in HER2+ breast cancer, we constructed ultrasound‐targeting nanobubbles (NT5E‐FITC‐NBs) based on senescence for ultrasound molecular‐targeted imaging.

Further, we developed a cellular senescence‐based ultrasound‐targeted NBs to dynamically monitor and effectively evaluate LAP resistance in HER2+ breast cancer in the present study. Ultrasound contrast agents (UCAs) are the basis and key of ultrasound molecular‐targeted imaging. Microbubbles are blood pool contrast agents with diameters ranging from 1 to 10 μm, which cannot pass through the tumor's leaky vasculature and remain in the circulation until they are taken up by the spleen and liver or dissolve in a rather short time.^38^ Due to these premises, nanoscale carriers can escape from capillaries and blood vessels and enter the defective tumor microcirculation to target tumor cells through enhanced permeability and retention (EPR) effects.^39^ In the present study, we assembled a nanoscale ultrasound‐targeted contrast agent (NT5E‐FITC‐NBs), in conjugation with specific senescence marker protein NT5E. With the advantage of nanoscale and active targeting of ligand NT5E, NT5E‐FITC‐NBs exhibits a good engagement with senescent tumor cells in the in vitro and in vivo settings. Targeted imaging could not only enhance the effect of ultrasound imaging, but also monitor the dynamic changes of cellular senescence after LAP mediated targeted therapy by observing the alteration in echo intensity, which was of great significance for evaluating drug resistance caused by cellular senescence.

## 5 LIMITATIONS

There are also limitations in this study. There are only one HER2+ cell line and one mice model used to study the correlation between cellular senescence and drug resistance. Additional HER2+ breast cancer cell lines would be used to provide a more comprehensive understanding of the role of cellular senescence in LAP resistance. The animal model used in this study was nude mice, which could not provide an immune environment for NT5E to exert suppressing anti‐tumor immune responses. In the future, we will use other animal models to explore more roles of NT5E in drug resistance. Finally, the targeted nanobubbles are still in the preclinical setting, so the feasibility and safety of clinical transformation still need to be further verified.

## 6 CONCLUSION

In summary, our work demonstrates that reversible cellular senescence contributes to LAP resistance in HER2+ breast cancer and identifies that NT5E can be an effective marker of cellular reversible senescence. We have devised ultrasound‐targeted nanobubbles NT5E‐FITC‐NBs with higher binding ability to reversible senescence cells and good imaging effects in B‐mode and CEUS detected by ultrasonic equipment. We have shown that cellular senescence‐based ultrasound‐targeted imaging could identify reversible senescence early, dynamically monitor and effectively evaluate LAP resistance in HER2+ breast cancer, which has the potential to improve cancer treatment outcomes by altering therapeutic strategies ahead of aggressive recurrences.

## AUTHOR CONTRIBUTIONS

**Xiaoyu Chen:** Conceptualization (equal); methodology (equal); writing – original draft (lead). **Ying Li:** Validation (equal); writing – original draft (equal). **Zhiwei Zhou:** Visualization (equal); writing – original draft (equal). **Yanqiu Zhang:** Investigation (equal); validation (equal). **Luchen Chang:** Formal analysis (equal); methodology (equal). **Xiujun Gao:** Resources (equal); validation (equal). **Qing Li:** Investigation (equal). **Hao Luo:** Validation (equal). **Kenneth D. Westover:** Methodology (equal). **Jialin Zhu:** Conceptualization (equal); writing – review and editing (equal). **Xi Wei:** Conceptualization (lead); funding acquisition (lead); project administration (lead); software (equal); supervision (lead); writing – review and editing (lead).

## FUNDING INFORMATION

The work was supported by the National Natural Science Foundation of China (81771852), Tianjin Major Science and Technology Project of Artificial Intelligence (18ZXZNSY00300), Tianjin Health Research Project (ZD20018 and QN20018), and Tianjin Research Innovation Project for Postgraduate Students (2020YJSS178).

## CONFLICT OF INTEREST STATEMENT

The authors declare that they have no known competing financial interests or personal relationships that could have appeared to influence the work reported in this paper.

## ETHICS STATEMENT

This protocol was approved by the Ethics Committee of Tianjin Medical University Cancer Institute and Hospital.

## PATIENT CONSENT STATEMENT

The patients' informed consent and signature were obtained.

## Supporting information


Appendix S1.

## Data Availability

All data generated or analyzed during this study are included in this published article or uploaded as supplementary information.
