# Supplementary material for: Dynamic ultrasound molecular‐targeted imaging of senescence in evaluation of lapatinib resistance in HER2‐positive breast cancer
Source: Cancer Med. 2023 Oct 4;12(19):19904–20. doi: 10.1002/cam4.6607 (PMC10587953; doi:10.1002/cam4.6607)
Supplement: Supplementary file 1 — Appendix S1. [file CAM4-12-19904-s001.docx]

**Methods**

**RNA-Seq**

**mRNA library construction and sequencing**

Total RNA was isolated and purified using TRIzol reagent (Invitrogen, Carlsbad, CA, USA) following the manufacturer's procedure. The RNA amount and purity of each sample was quantified using NanoDrop ND-1000 (NanoDrop, Wilmington, DE, USA). The RNA integrity was assessed by Bioanalyzer 2100 (Agilent, CA, USA) with RIN number >7.0, and confirmed by electrophoresis with denaturing agarose gel. Poly (A) RNA is purified from 1μg total RNA using Dynabeads Oligo (dT)25-61005 (Thermo Fisher, CA, USA) using two rounds of purification. Then the poly(A) RNA was fragmented into small pieces using Magnesium RNA Fragmentation Module (NEB, cat. e6150, USA) under 94℃ 5-7min. Then the cleaved RNA fragments were reverse-transcribed to create the cDNA by SuperScript™ II Reverse Transcriptase (Invitrogen, cat. 1896649, USA), which were next used to synthesise U-labeled second-stranded DNAs with E. coli DNA polymerase I (NEB, cat. m0209, USA), RNase H (NEB, cat. m0297, USA) and dUTP Solution (Thermo Fisher, cat. R0133, USA）. An A-base is then added to the blunt ends of each strand, preparing them for ligation to the indexed adapters. Each adapter contains a T-base overhang for ligating the adapter to the A-tailed fragmented DNA. Single- or dual-index adapters are ligated to the fragments, and size selection was performed with AMPureXP beads. After the heat-labile UDG enzyme (NEB, cat. m0280, USA) treatment of the U-labeled second-stranded DNAs, the ligated products are amplified with PCR by the following conditions: initial denaturation at 95℃ for 3 min; 8 cycles of denaturation at 98℃ for 15 sec, annealing at 60℃ for 15 sec, and extension at 72℃ for 30 sec; and then final extension at 72℃ for 5 min. The average insert size for the final cDNA library was 300±50 bp. At last, we performed the 2×150bp paired-end sequencing (PE150) on an Illumina Novaseq™ 6000 (LC-Bio Technology CO., Ltd., Hangzhou, China) following the vendor's recommended protocol.

**Sequence and primary analysis:**

Cutadapt software (<https://cutadapt.readthedocs.io/en/stable/,version:cutadapt-1.9>) was used to remove the reads that contained adaptor contamination, (command line: ~cutadapt -a ADAPT1 -A ADAPT2 -o out1. fastq -p out2. fastq in1. fastq in2. fastq -O 5 -m 100). And After removed the low quality bases and undetermined bases ,we used HISAT2 software (<https://daehwankimlab.github.io/hisat2/,version:hisat2-2.0.4>) to map reads to the genome (for example:Homo sapiens Ensembl v96), (command line: ~hisat2 -1 R1.fastq.gz -2 R1.fastq.gz -S sample_mapped.sam). The mapped reads of each sample were assembled using StringTie (<http://ccb.jhu.edu/software/stringtie/,version:stringtie-1.3.4d.Linux_x86_64>) with default parameters (command line: ~stringtie -p 4 -G genome. gtf -o output. gtf -l sample input.bam). Then, all transcriptomes from all samples were merged to reconstruct a comprehensive transcriptome using gffcompare software(<http://ccb.jhu.edu/software/stringtie/gffcompare.shtml,version:gffcompare-0.9.8.Linux_x86_64>).After the final transcriptome was generated, StringTie and ballgown(<http://www.bioconductor.org/packages/release/bioc/html/ballgown.html>) were used to estimate the expression levels of all transcripts and perform expression level for mRNAs by calculating FPKM (FPKM=[total_exon_fragments / mapped_reads(millions) × exon_length(kB)]),(command line: ~stringtie -e -B -p 4 -G merged. gtf -o samples. gtf samples.bam). The differentially expressed mRNAs were selected with fold change > 2 or fold change < 0.5 and p value < 0.05 by R package edgeR(<https://bioconductor.org/packages/release/bioc/html/edgeR.html>) or DESeq2(<http://www.bioconductor.org/packages/release/bioc/html/DESeq2.html>), and then analysis GO enrichment and KEGG enrichment to the differentially expressed mRNAs.

**Supplemental Figures**

**
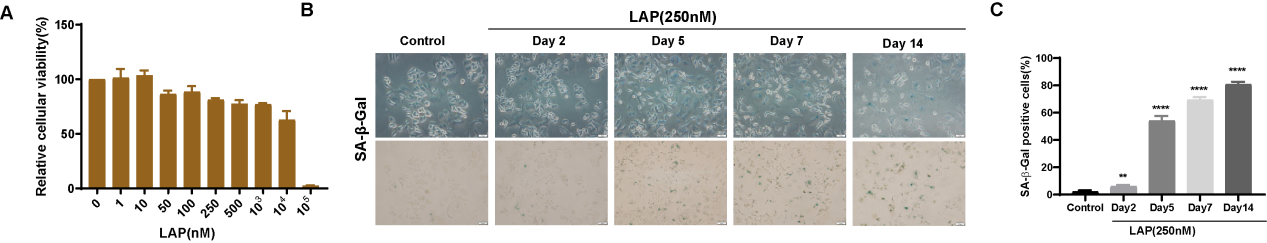
**

**Figure S1.** (A) The cell proliferation was assessed using CCK-8 assay. (B and C) Growth of this cell line over a 2-week period in 250 nM LAP caused an accumulation of senescent cells until 80% of cells were senescent by day 7. LAP: lapatinib.**p*<0.05, ***p*<0.01, ****p*<0.001. Data are expressed as Mean ± SD (n=3).

**
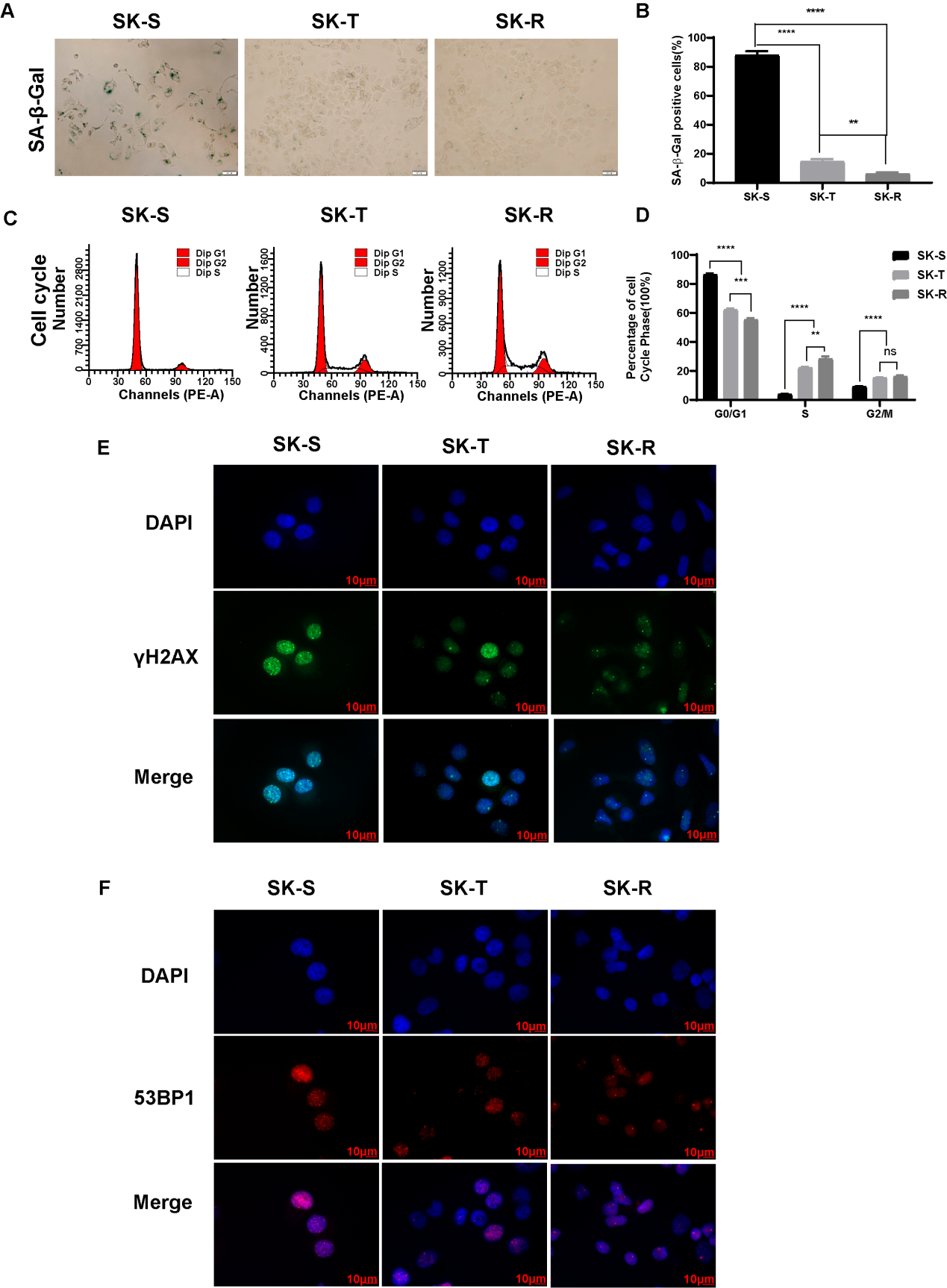
**

**Figure S2.** (A and B) In comparison to SK-S cells, the SA-β-Gal staining of SK-T and SK-R was negative. (C and D) The cell population in the G0/G1 phase was marked decreased in SK-T and SK-R cells. (E and F) The IF staining showed a remarkable decline in the protein expression level of γH2AX and 53BP1 in DNA damage foci of SK-T and SK-R cells(scale bar: 10 μm). SK-S: senescent SKBR3 cells; SK-T: senescence escaped SKBR3 cells; SK-R: LAP-resistant cells; LAP: lapatinib. **p*<0.05, ***p*<0.01, ****p*<0.001. Data are expressed as Mean ± SD (n=3).


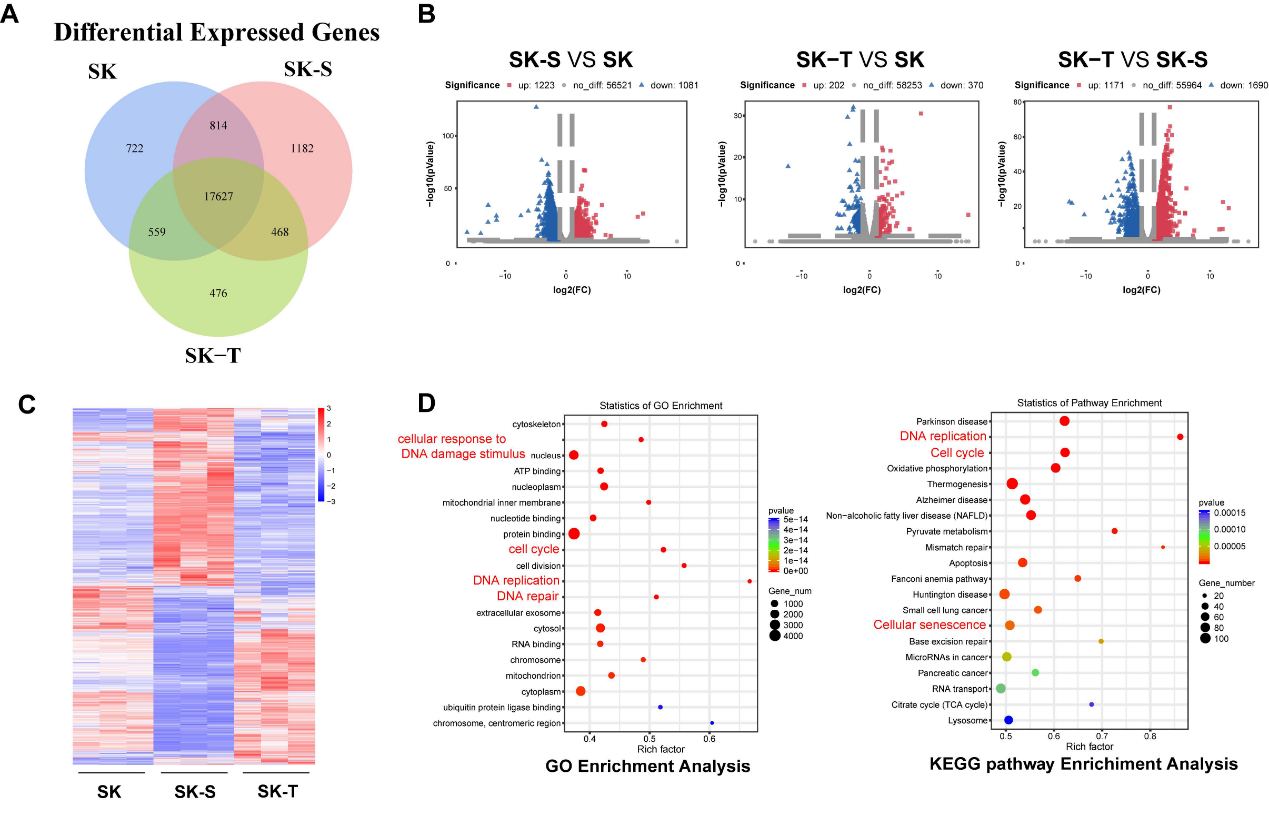


**Figure S3.** (A) We performed RNA-seq to evaluate the alterations in gene expression on three groups of cells. There were 722 unique genes for SK cells, 1,182 unique genes for SK-S cells, and 476 unique genes for SK-T cells. (B) Further analysis for changes in the expression level revealed 1,223 upregulated genes and 1,081 downregulated genes in SK-S compared to SK cells. (C) The exhibition on the heat map presented a total number of 7,283 genes that differed significantly among the three groups of cells. (D) GO enrichment analysis and KEGG enrichment analysis were carried out for differentially expressed genes in the three groups of cells, respectively. SK: parental SKBR3 cells; SK-S: senescent SKBR3 cells; SK-T: senescence escaped SKBR3 cells. Data are expressed as Mean ± SD (n=3).


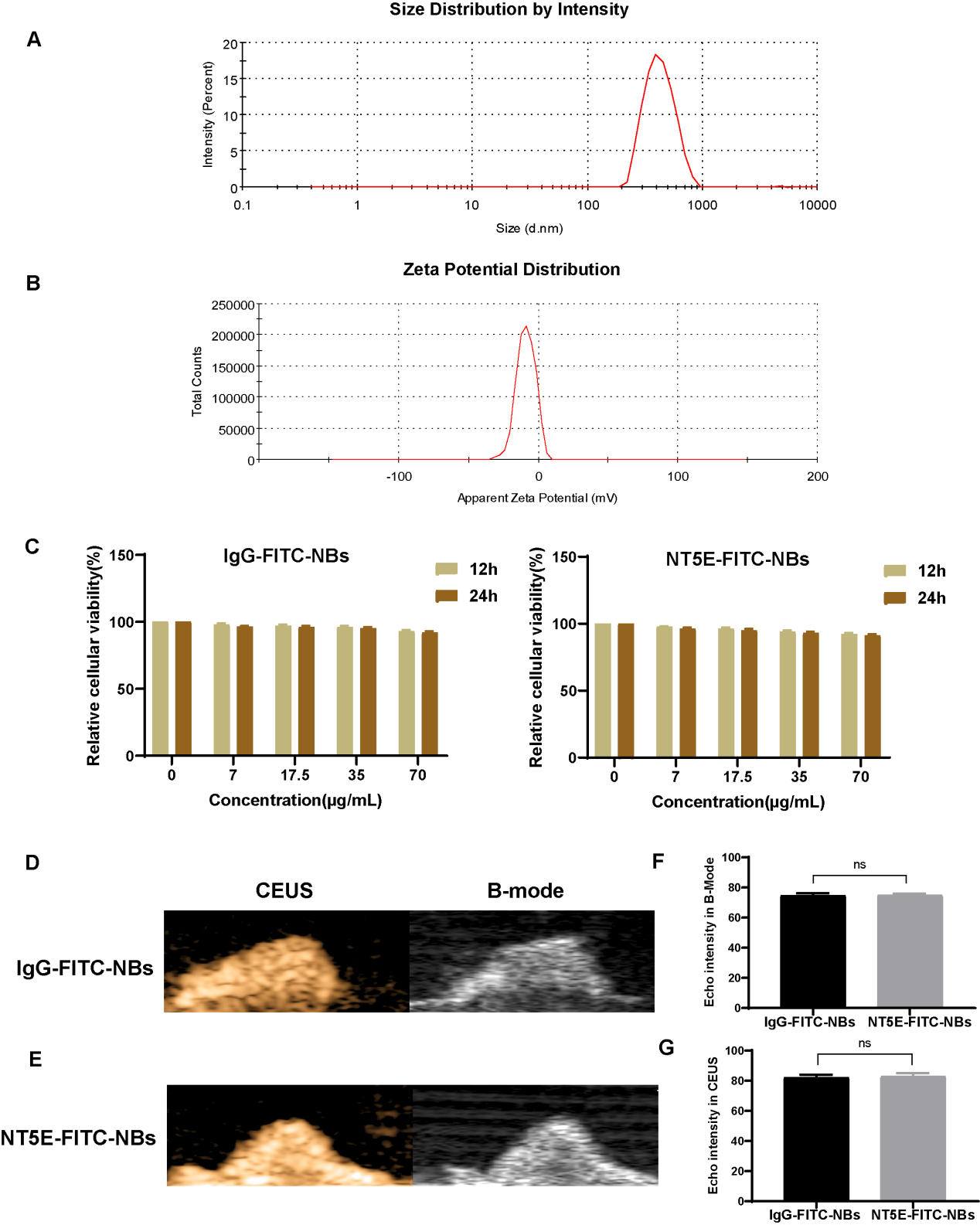


**Figure S4.** (A and B) Then IgG antibody labeled with green fluorescence was coupled to the nanobubble shell to obtain IgG-FITC-NBs. (C) IgG-FITC-NBs and NT5E-FITC-NBs with different concentrations were incubated with SK cells for 12 and 24 hours, respectively, and it was found that there was no statistical difference in cell activity under different concentrations of NBs. (D-G) The *in vitro* imaging effect of NT5E-FITC-NBs and IgG-FITC-NBs detected by ultrasonic equipment. SK: parental SKBR3 cells; Data are expressed as Mean ± SD (n=3).

**
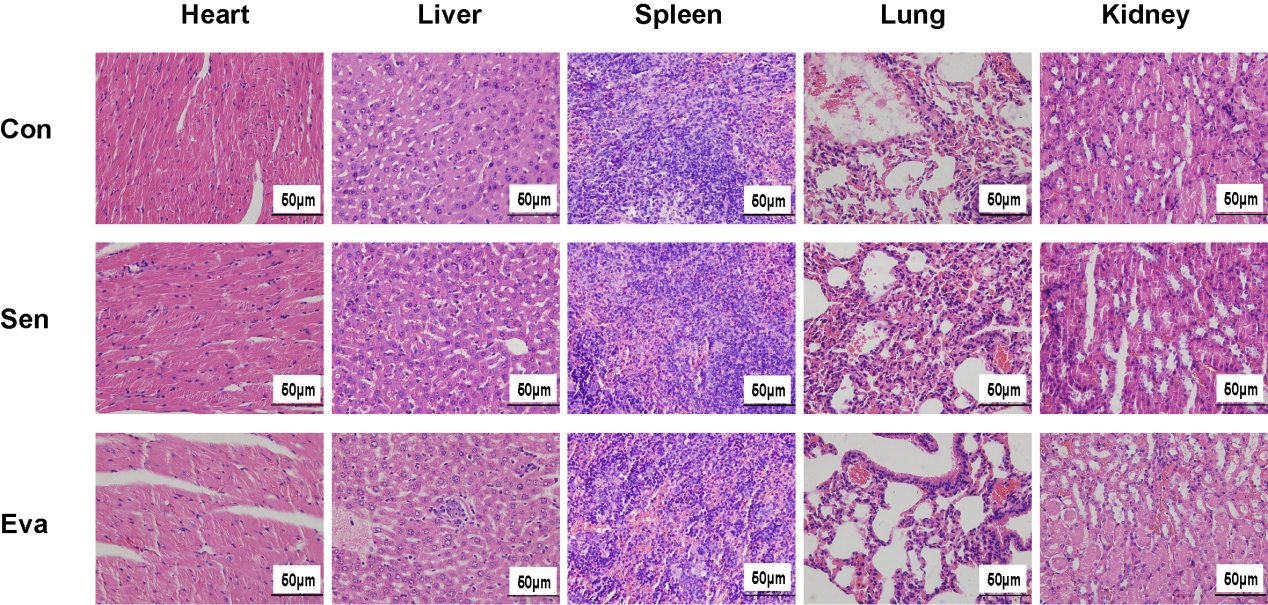
**

**Figure S5.** In vivo biosafety analysis of NT5E-FITC-NBs. Viscera (heart, liver, spleen, lung, and kidney) was taken from the mice for HE staining. It was found that there was no significant difference in morphology and structure of the heart, liver, spleen, lung, and kidney of the three groups of mice in HE staining (scale bar: 50 μm).

**Table S1**. Real-time quantitative primers sequences used in this study

| P15 | Forward Primer | GGACTAGTGGAGAAGGTGC |
| --- | --- | --- |
|  | Reverse Primer | CCCATCATCATGACCTGGATC |
| P16 | Forward Primer | CCGTGGACCTGGCTGAGGAG |
|  | Reverse Primer | CGGGGATGTCTGAGGGACCTTC |
| P21 | Forward Primer | TGTCCGTCAGAACCCATGC |
|  | Reverse Primer | AAAGTCGAAGTTCCATCGCTC |
| P27 | Forward Primer | AACGTGCGAGTGTCTAACGG |
|  | Reverse Primer | CCCTCTAGGGGTTTGTGATTCT |
| P53 | Forward Primer | CAGCACATGACGGAGGTTGT |
|  | Reverse Primer | TCATCCAAATACTCCACACGC |
